# Supplementary figures and images for: Increased risk of falls and fractures in patients with psychosis and Parkinson disease
Source: PLoS One. 2021 Jan 27;16(1):e0246121. doi: 10.1371/journal.pone.0246121 (PMC7840029; doi:10.1371/journal.pone.0246121)

S1 Fig. Assignment of the cohort eligibility date


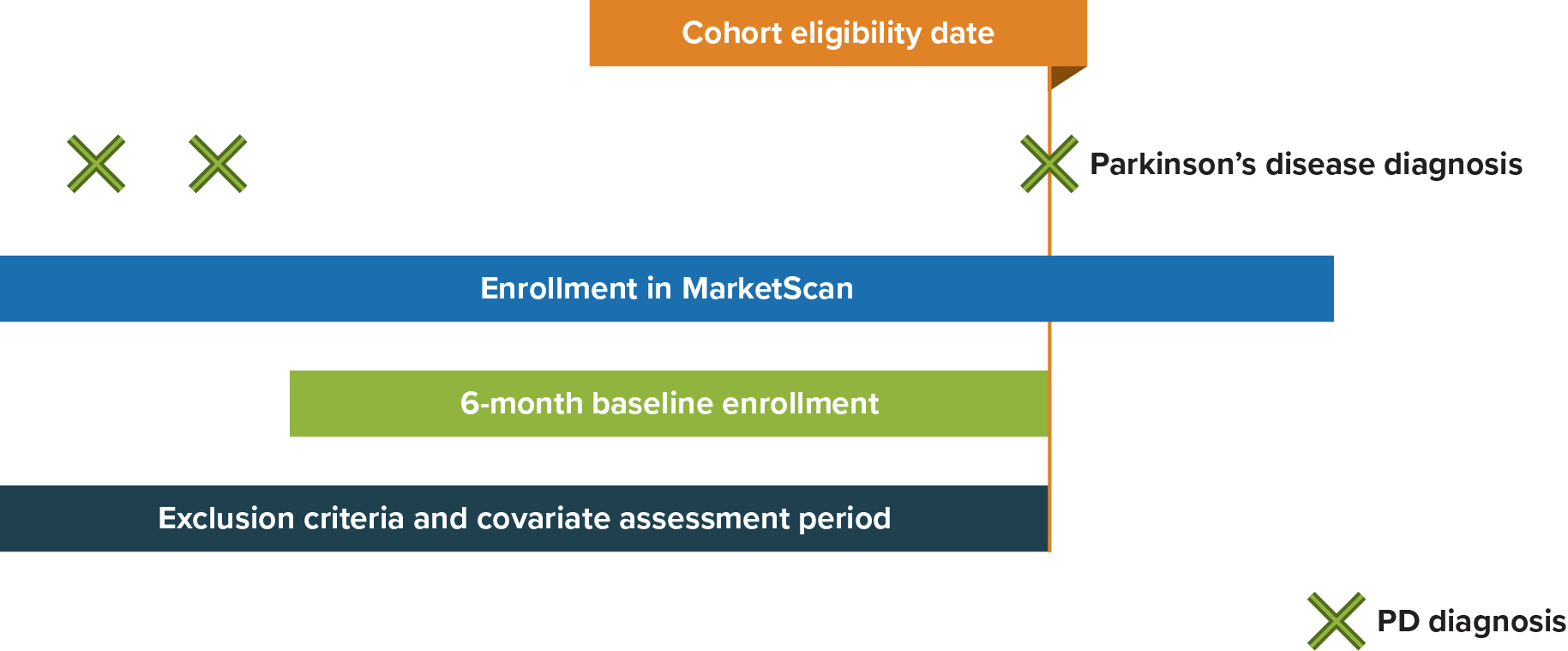


PD = Parkinson disease.

Supplement: S1 Fig — PD = Parkinson disease. (DOCX) [file pone.0246121.s001.docx]
