# Supplementary material for: Increased risk of falls and fractures in patients with psychosis and Parkinson disease
Source: PLoS One. 2021 Jan 27;16(1):e0246121. doi: 10.1371/journal.pone.0246121 (PMC7840029; doi:10.1371/journal.pone.0246121)
Supplement: S2 Fig — PD = Parkinson disease; PDP = Parkinson disease with psychosis. (DOCX) [file pone.0246121.s002.docx]

S2 Fig. Assignment of person-time to the Parkinson disease with and without psychosis groups relative to the PD cohort eligibility date and the PDP index date


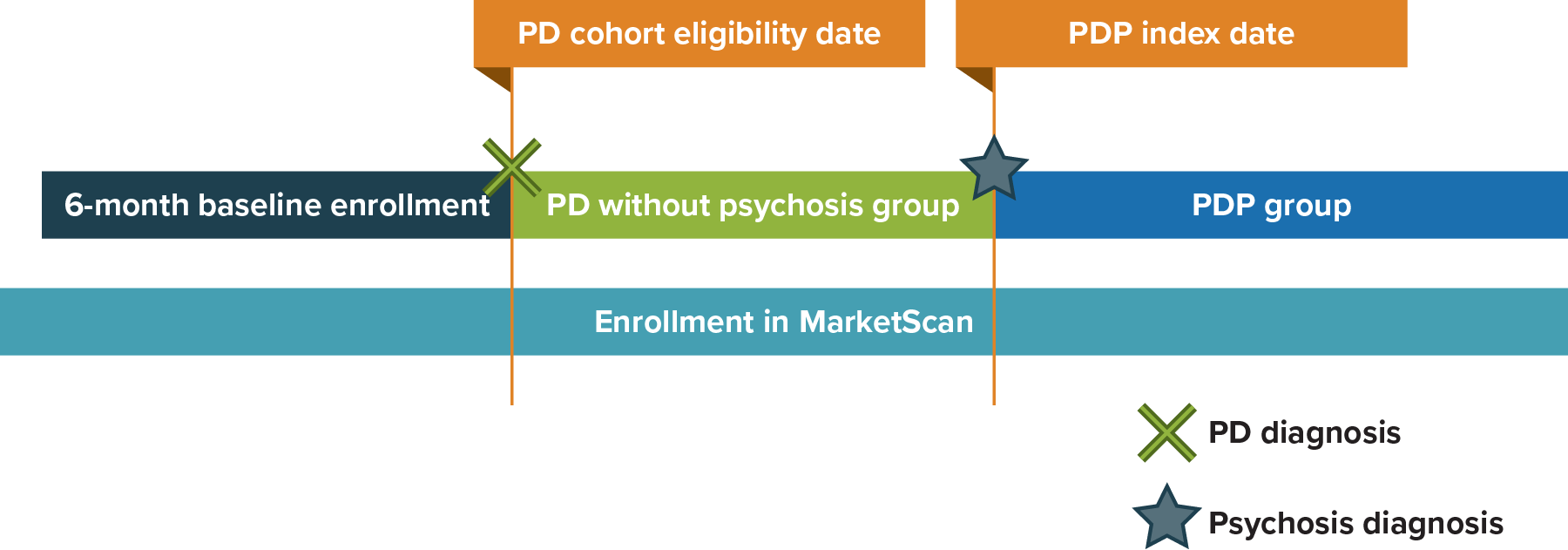


PD = Parkinson disease; PDP = Parkinson disease with psychosis.
