# Supplementary material for: Increased risk of falls and fractures in patients with psychosis and Parkinson disease
Source: PLoS One. 2021 Jan 27;16(1):e0246121. doi: 10.1371/journal.pone.0246121 (PMC7840029; doi:10.1371/journal.pone.0246121)
Supplement: S1 Methods — (DOCX) [file pone.0246121.s003.docx]

# S1 Methods

## Sequential propensity score matching approach

We created 4-month blocks of follow-up time since the PD cohort eligibility date for all patients in the overall PD group (all time after 7 years from the PD cohort eligibility date was collapsed into a single block).

Time blocks and potential index date since the Parkinson disease cohort eligibility date, unmatched cohort

**
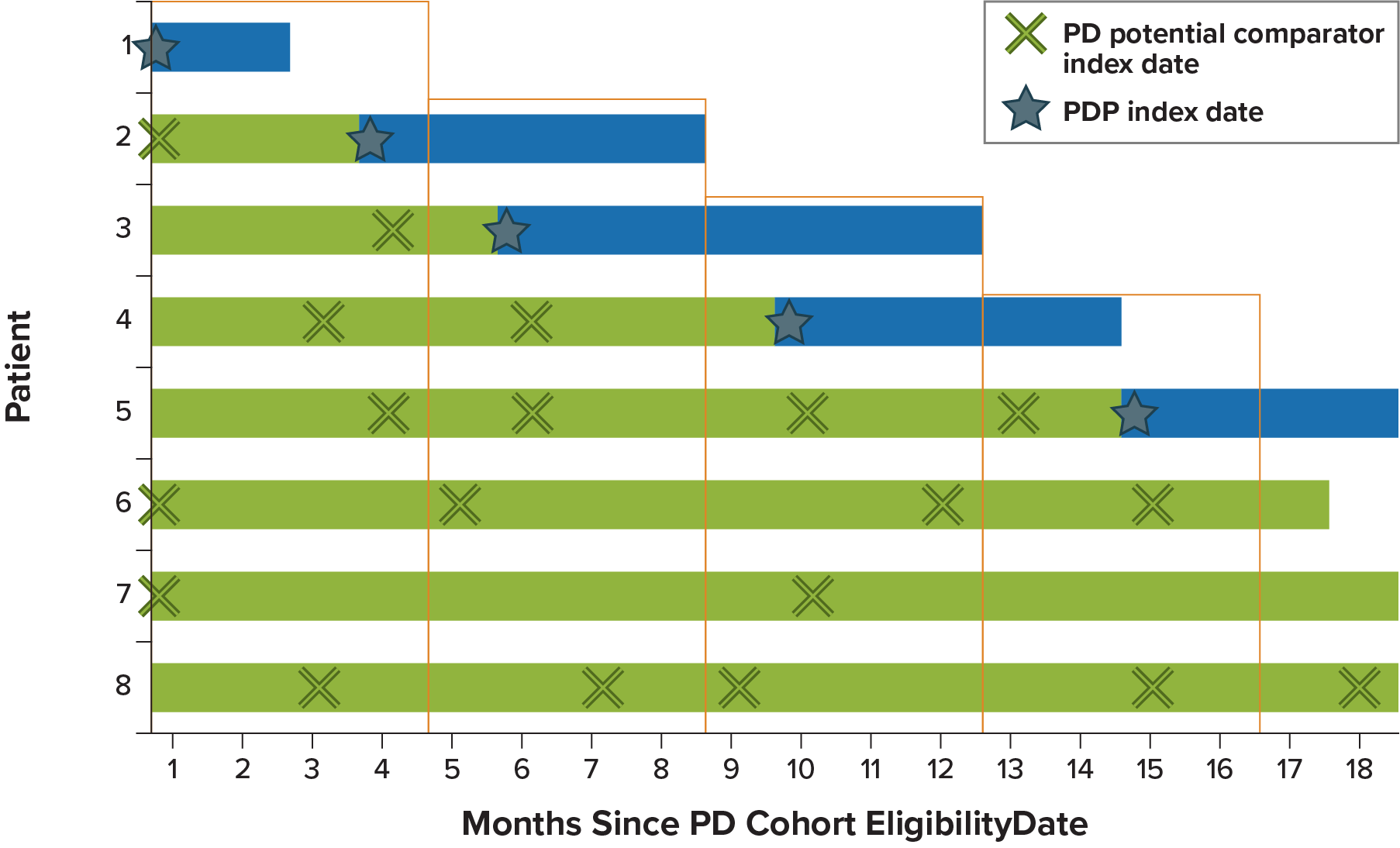
**

PD = Parkinson disease; PDP = Parkinson disease with psychosis.

All patients with PDP index dates within a given block were included in the block-specific propensity score model as the exposed treatment group. The unexposed treatment group within each time block consisted of patients without PDP (or prior to a PDP diagnosis) who had a claim with a PD diagnosis occurring during the time block; this might be the index PD diagnosis or any recorded diagnosis code occurring during non-PDP person-time. The date of the PD diagnosis in the block was considered the potential comparator index date, and the patient’s covariates were reassessed on that potential index date. For those with multiple PD diagnoses during the block, a randomly selected date of one of the patient’s PD diagnosis during the block was used. Within each block, a multivariable logistic regression model was used to estimate block-specific propensity scores using the covariates described in previous sections; a covariate could be dropped from a block-specific propensity score model if, in a given block, the prevalence of a covariate was 0% or 100% in one of the groups. After the block-specific propensity scores were estimated, PD without psychosis comparator index dates were matched to the PDP index dates with 2:1 fixed-ratio matching using a greedy, nearest neighbor 5- to 1-digit matching algorithm, without replacement (Parsons, 2004). A maximum caliper of 0.2 times the SD of the estimated logit of the propensity score was used (Austin, 2011).

Separate propensity score models were constructed in each time block, and a patient’s covariates were updated at each date and used in each block. If a potential PD index date from a patient matched in one time block, then potential PD index dates from the same patient were not considered in subsequent blocks. However, these individuals were eligible to qualify as patients with PDP during follow-up if new-onset psychosis diagnosis was subsequently observed. All the resulting matches from each block-specific assessment were compiled into the final, matched cohort with the PDP index dates and matched PD index dates used as the matched cohort index dates.

Time blocks and matched index date in the propensity score-matched PD-PDP cohort


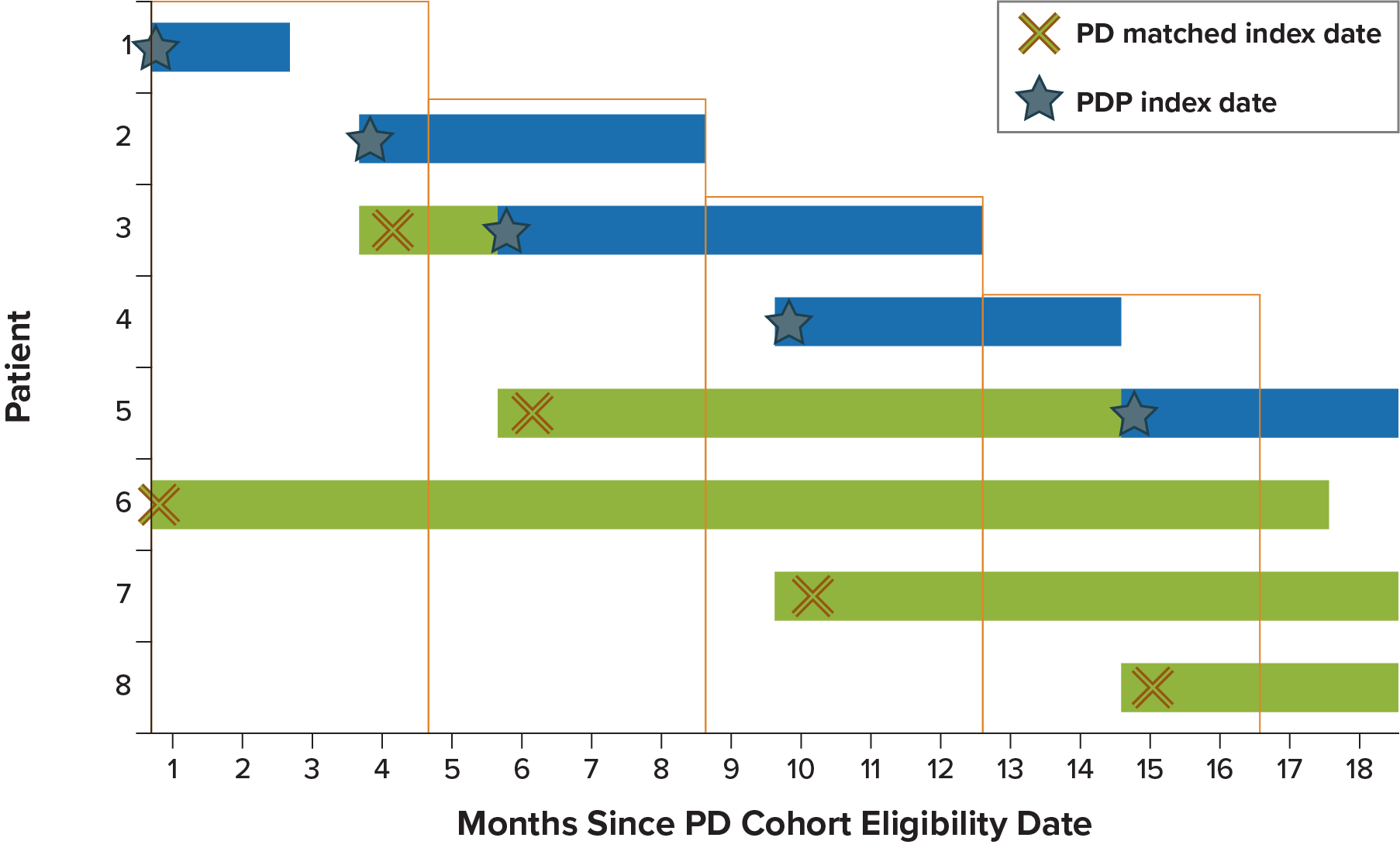


PD = Parkinson disease; PDP = Parkinson disease with psychosis.
