# Supplementary material for: Increased risk of falls and fractures in patients with psychosis and Parkinson disease
Source: PLoS One. 2021 Jan 27;16(1):e0246121. doi: 10.1371/journal.pone.0246121 (PMC7840029; doi:10.1371/journal.pone.0246121)
Supplement: S1 Table — CI = confidence interval; IR, incidence rate; PD = Parkinson disease; PDP = Parkinson disease with psychosis. a All patients with PD who did not have a first psychosis diagnosis on their PD eligibility date. b Patients who met the criteria to enter the PDP cohort. c The number of events was assumed to follow a Poisson distribution; corresponding exact 95% CIs were computed using methods described in Dobson et al. (1991). (DOCX) [file pone.0246121.s004.docx]

S1 Table. Crude incidence rates of falls and fractures for the unmatched PD without psychosis a and PDP b groups, overall and by time interval

| Outcome in specified time period | Group | No. of patients | No. of events | No. of person-years | IR (95% CI) c per 100 person-years |
| --- | --- | --- | --- | --- | --- |
| Composite falls/fractures | |  |  |  |  |
| Overall | PDP | 12,127 | 5,453 | 18,783 | 29.03 (28.27-29.81) |
|  | PD | 154,306 | 36,341 | 318,488 | 11.41 (11.29-11.53) |
| 0-1 year | PDP | 12,127 | 2,925 | 8,587 | 34.06 (32.84-35.32) |
|  | PD | 154,306 | 13,471 | 120,925 | 11.14 (10.95-11.33) |
| > 1 year-2 years | PDP | 6,275 | 1,088 | 4,784 | 22.74 (21.41-24.14) |
|  | PD | 93,663 | 7,758 | 75,665 | 10.25 (10.03-10.48) |
| > 2 years-3 years | PDP | 3,571 | 671 | 2,723 | 24.64 (22.81-26.58) |
|  | PD | 59,346 | 5,055 | 48,390 | 10.45 (10.16-10.74) |
| > 3 years-4 years | PDP | 2,010 | 403 | 1,462 | 27.57 (24.95-30.40) |
|  | PD | 38,293 | 3,541 | 30,653 | 11.55 (11.17-11.94) |
| > 4 years | PDP | 1,014 | 366 | 1,228 | 29.81 (26.83-33.02) |
|  | PD | 23,980 | 6,516 | 42,854 | 15.21 (14.84-15.58) |
| Falls |  |  |  |  |  |
| Overall | PDP | 12,128 | 4,876 | 18,795 | 25.94 (25.22-26.68) |
|  | PD | 154,314 | 29,266 | 318,622 | 9.19 (9.08-9.29) |
| 0-1 year | PDP | 12,128 | 2,614 | 8,594 | 30.42 (29.26-31.61) |
|  | PD | 154,314 | 10,557 | 120,982 | 8.73 (8.56-8.89) |
| > 1 year-2 years | PDP | 6,275 | 962 | 4,786 | 20.10 (18.85-21.41) |
|  | PD | 93,663 | 6,132 | 75,695 | 8.10 (7.90-8.31) |
| > 2 years-3 years | PDP | 3,571 | 591 | 2,724 | 21.69 (19.98-23.51) |
|  | PD | 59,346 | 3,998 | 48,410 | 8.26 (8.00-8.52) |
| > 3 years-4 years | PDP | 2,010 | 375 | 1,462 | 25.65 (23.12-28.38) |
|  | PD | 38,293 | 2,875 | 30,666 | 9.38 (9.04-9.72) |
| > 4 years | PDP | 1,014 | 334 | 1,229 | 27.19 (24.35-30.27) |
|  | PD | 23,980 | 5,704 | 42,870 | 13.31 (12.96-13.66) |
| Any fracture |  |  |  |  |  |
| Overall | PDP | 12,131 | 943 | 18,872 | 5.00 (4.68-5.33) |
|  | PD | 154,325 | 10,155 | 318,985 | 3.18 (3.12-3.25) |
| 0-1 year | PDP | 12,131 | 501 | 8,636 | 5.80 (5.30-6.33) |
|  | PD | 154,325 | 4,078 | 121,106 | 3.37 (3.26-3.47) |
| > 1 year-2 years | PDP | 6,275 | 220 | 4,800 | 4.58 (4.00-5.23) |
|  | PD | 93,665 | 2,250 | 75,769 | 2.97 (2.85-3.09) |
| > 2 years-3 years | PDP | 3,571 | 113 | 2,733 | 4.13 (3.41-4.97) |
|  | PD | 59,347 | 1,479 | 48,457 | 3.05 (2.90-3.21) |
| > 3 years-4 years | PDP | 2,010 | 56 | 1,468 | 3.81 (2.88-4.95) |
|  | PD | 38,293 | 958 | 30,702 | 3.12 (2.93-3.32) |
| > 4 years | PDP | 1,014 | 53 | 1,234 | 4.30 (3.22-5.62) |
|  | PD | 23,981 | 1,390 | 42,951 | 3.24 (3.07-3.41) |

CI = confidence interval; IR, incidence rate; PD = Parkinson disease; PDP = Parkinson disease with psychosis.

a All patients with PD who did not have a first psychosis diagnosis on their PD eligibility date.

b Patients who met the criteria to enter the PDP cohort.

c The number of events was assumed to follow a Poisson distribution; corresponding exact 95% CIs were computed using methods described in Dobson et al. (1991).
