# Supplementary material for: Increased risk of falls and fractures in patients with psychosis and Parkinson disease
Source: PLoS One. 2021 Jan 27;16(1):e0246121. doi: 10.1371/journal.pone.0246121 (PMC7840029; doi:10.1371/journal.pone.0246121)
Supplement: S2 Table — CI = confidence interval; IR, incidence rate; PD = Parkinson disease; PDP = Parkinson disease with psychosis. a All patients with PD who did not have a first psychosis diagnosis on their PD eligibility date. b Patients who met the criteria to enter the PDP cohort. c The number of events was assumed to follow a Poisson distribution; corresponding exact 95% CIs were computed using methods described in Dobson et al. (1991). (DOCX) [file pone.0246121.s005.docx]

S2 Table. Incidence rates of falls and fractures for the unmatched PD without psychosis ^a^ and PDP^b^ groups, by age category at index date

| Outcome in specified age category | Group | No. of patients | No. of events | No. of person-years | IR (95% CI) c per 100 person-years |
| --- | --- | --- | --- | --- | --- |
| 40 to < 65 years old |  |  |  |  |  |
| Composite falls/fractures | PDP | 1,206 | 333 | 1,856 | 17.94 (16.06-19.97) |
|  | PD | 45,676 | 5,083 | 97,978 | 5.19 (5.05-5.33) |
| Falls | PDP | 1,206 | 297 | 1,857 | 15.99 (14.23-17.92) |
|  | PD | 45,676 | 3,850 | 98,002 | 3.93 (3.81-4.05) |
| Any fracture | PDP | 1,206 | 56 | 1,862 | 3.01 (2.27-3.91) |
|  | PD | 45,675 | 1,644 | 98,044 | 1.68 (1.60-1.76) |
| 65 to < 70 years old |  |  |  |  |  |
| Composite falls/fractures | PDP | 839 | 357 | 1,603 | 22.27 (20.02-24.71) |
|  | PD | 15,436 | 3,354 | 36,012 | 9.31 (9.00-9.63) |
| Falls | PDP | 839 | 323 | 1,603 | 20.14 (18.01-22.46) |
|  | PD | 15,436 | 2,690 | 36,025 | 7.47 (7.19-7.75) |
| Any fracture | PDP | 840 | 49 | 1,609 | 3.05 (2.25-4.03) |
|  | PD | 15,438 | 924 | 36,058 | 2.56 (2.40-2.73) |
| 70 to < 85 years old |  |  |  |  |  |
| Composite falls/fractures | PDP | 6,893 | 3,482 | 11,279 | 30.87 (29.85-31.91) |
|  | PD | 70,279 | 20,962 | 148,917 | 14.08 (13.89-14.27) |
| Falls | PDP | 6,894 | 3,108 | 11,287 | 27.54 (26.58-28.52) |
|  | PD | 70,283 | 16,945 | 148,993 | 11.37 (11.20-11.55) |
| Any fracture | PDP | 6,894 | 625 | 11,335 | 5.51 (5.09-5.96) |
|  | PD | 70,288 | 5,782 | 149,205 | 3.88 (3.78-3.98) |
| ≥ 85 years old |  |  |  |  |  |
| Composite falls/fractures | PDP | 3,189 | 1,281 | 4,045 | 31.67 (29.96-33.45) |
|  | PD | 22,915 | 6,942 | 35,580 | 19.51 (19.05-19.98) |
| Falls | PDP | 3,189 | 1,148 | 4,048 | 28.36 (26.75-30.05) |
|  | PD | 22,919 | 5,781 | 35,602 | 16.24 (15.82-16.66) |
| Any fracture | PDP | 3,191 | 213 | 4,066 | 5.24 (4.56-5.99) |
|  | PD | 22,924 | 1,805 | 35,678 | 5.06 (4.83-5.30) |

CI = confidence interval; IR, incidence rate; PD = Parkinson disease; PDP = Parkinson disease with psychosis.

a All patients with PD who did not have a first psychosis diagnosis on their PD eligibility date.

b Patients who met the criteria to enter the PDP cohort.

c The number of events was assumed to follow a Poisson distribution; corresponding exact 95% CIs were computed using methods described in Dobson et al. (1991).
