# Supplementary material for: Increased risk of falls and fractures in patients with psychosis and Parkinson disease
Source: PLoS One. 2021 Jan 27;16(1):e0246121. doi: 10.1371/journal.pone.0246121 (PMC7840029; doi:10.1371/journal.pone.0246121)
Supplement: S3 Table — HIV/AIDS = human immunodeficiency virus/acquired immune deficiency syndrome; PD = Parkinson disease; PDP = Parkinson disease with psychosis; SD = standard deviation. Note: All characteristics were assessed during the entire look-back period unless otherwise stated. a Patients with PD without psychosis who were selected for the matched cohort; evaluated at the index date of the matched PD diagnosis. b Patients who met the criteria to enter the PDP cohort; evaluated at their first psychosis diagnosis date. c Patients who did not develop psychosis at the PD cohort eligibility date and were not selected for the matched cohort. Evaluated at their PD cohort eligibility date. d Assessed in a look-back period of up to 1 year before the corresponding cohort entry/eligibility date. e Comprised all systemic glucocorticoids (excluded nonsystemic administration routes such as topical or inhaled applications). f Comprised levodopa-carbidopa, anticholinergics, dopamine agonists, monoamine oxidase B inhibitors, and catechol-O-methyltransferase inhibitors. g Assessed in the 6 months before the corresponding cohort entry/eligibility date. (DOCX) [file pone.0246121.s006.docx]

S3 Table. Descriptive characteristics of patients with Parkinson disease with and without psychosis, matched cohort and excluded from matched cohort

| **Demographic** | **Matched cohort** | | | **Excluded from matched cohort** | | |
| --- | --- | --- | --- | --- | --- | --- |
|  | **PD without psychosis^a^ (N = 24,164)** | **PDP^b^ (N = 12,082)** | **Standardized mean difference** | **PD without psychosis^c^ (N = 130,175)** | **PDP^b^ (N = 50)** | **Standardized mean difference** |
| Age at index, mean (SD) | 78.4 (9.00) | 78.1 (9.24) | 0.03 | 71.3 (11.75) | 81.7 (7.72) | −1.04 |
| Sex, female, n (%) | 9,599 (39.7) | 4,747 (39.3) | 0.01 | 51,873 (39.8) | 26 (52.0) | −0.25 |
| Frailty indicators, n (%) |  |  |  |  |  |  |
| Ambulance/life support | 14,180 (58.7) | 7,024 (58.1) | 0.01 | 25,126 (19.3) | 49 (98.0) | −2.66 |
| Arthritis | 16,899 (69.9) | 8,380 (69.4) | 0.01 | 58,988 (45.3) | 47 (94.0) | −1.25 |
| Bladder dysfunction | 8,124 (33.6) | 4,003 (33.1) | 0.01 | 18,170 (14.0) | 35 (70.0) | −1.38 |
| Cancer screening | 6,995 (28.9) | 3,556 (29.4) | −0.01 | 37,161 (28.5) | 14 (28.0) | 0.01 |
| Coagulopathy | 1,768 (7.3) | 905 (7.5) | −0.01 | 3,821 (2.9) | 12 (24.0) | −0.65 |
| Dementia | 14,946 (61.9) | 7,372 (61.0) | 0.02 | 32,059 (24.6) | 44 (88.0) | −1.66 |
| Diabetes mellitus complications | 3,577 (14.8) | 1,797 (14.9) | 0.00 | 10,716 (8.2) | 14 (28.0) | −0.53 |
| Difficulty walking | 14,170 (58.6) | 7,024 (58.1) | 0.01 | 35,965 (27.6) | 43 (86.0) | −1.46 |
| Heart failure | 8,249 (34.1) | 4,163 (34.5) | −0.01 | 19,574 (15.0) | 38 (76.0) | −1.55 |
| Home hospital bed | 2,026 (8.4) | 1,014 (8.4) | 0.00 | 3,527 (2.7) | 10 (20.0) | −0.57 |
| Home oxygen | 1,500 (6.2) | 738 (6.1) | 0.00 | 4,025 (3.1) | 11 (22.0) | −0.60 |
| Lipid abnormality | 14,767 (61.1) | 7,425 (61.5) | −0.01 | 62,105 (47.7) | 36 (72.0) | −0.51 |
| Paralysis | 1,981 (8.2) | 962 (8.0) | 0.01 | 4,717 (3.6) | 5 (10.0) | −0.26 |
| Podiatric care | 6,168 (25.5) | 3,085 (25.5) | 0.00 | 15,897 (12.2) | 24 (48.0) | −0.85 |
| Rehabilitation services | 7,532 (31.2) | 3,770 (31.2) | 0.00 | 16,307 (12.5) | 29 (58.0) | −1.08 |
| Sepsis | 8,450 (35.0) | 4,198 (34.7) | 0.00 | 20,291 (15.6) | 31 (62.0) | −1.08 |
| Skin ulcer | 4,203 (17.4) | 2,115 (17.5) | 0.00 | 8,103 (6.2) | 22 (44.0) | −0.97 |
| Stroke/brain injury | 7,928 (32.8) | 3,975 (32.9) | 0.00 | 15,587 (12.0) | 40 (80.0) | −1.87 |
| Vertigo | 8,029 (33.2) | 4,009 (33.2) | 0.00 | 21,522 (16.5) | 29 (58.0) | −0.95 |
| Weakness | 8,570 (35.5) | 4,310 (35.7) | 0.00 | 17,604 (13.5) | 36 (72.0) | −1.47 |
| Wheelchair use | 2,824 (11.7) | 1,417 (11.7) | 0.00 | 5,099 (3.9) | 13 (26.0) | −0.65 |
| Components of Charlson Comorbidity Index, n (%) |  |  |  |  |  |  |
| Chronic kidney disease | 4,176 (17.3) | 2,093 (17.3) | 0.00 | 10,116 (7.8) | 18 (36.0) | −0.73 |
| Chronic obstructive pulmonary disease | 6,960 (28.8) | 3,457 (28.6) | 0.00 | 21,232 (16.3) | 24 (48.0) | −0.72 |
| Diabetes mellitus | 7,862 (32.5) | 3,941 (32.6) | 0.00 | 30,447 (23.4) | 30 (60.0) | −0.80 |
| Hemiplegia | 1,405 (5.8) | 682 (5.6) | 0.01 | 3,469 (2.7) | 3 (6.0) | −0.16 |
| HIV/AIDS | 25 (0.1) | 9 (0.1) | 0.01 | 125 (0.1) | 1 (2.0) | −0.19 |
| Leukemia/lymphoma | 590 (2.4) | 280 (2.3) | 0.01 | 1,918 (1.5) | 1 (2.0) | −0.04 |
| Liver disease | 799 (3.3) | 411 (3.4) | −0.01 | 3,009 (2.3) | 2 (4.0) | −0.10 |
| Myocardial infarction | 2,534 (10.5) | 1,270 (10.5) | 0.00 | 5,619 (4.3) | 13 (26.0) | −0.63 |
| Peptic ulcer disease | 793 (3.3) | 426 (3.5) | −0.01 | 2,153 (1.7) | 5 (10.0) | −0.36 |
| Peripheral vascular disease | 8,376 (34.7) | 4,146 (34.3) | 0.01 | 21,064 (16.2) | 33 (66.0) | −1.17 |
| Tumor | 4,849 (20.1) | 2,471 (20.5) | −0.01 | 17,048 (13.1) | 15 (30.0) | −0.42 |
| Other predictors of falls or fractures, n (%) |  |  |  |  |  |  |
| Ankylosing spondylitis | 768 (3.2) | 378 (3.1) | 0.00 | 2,277 (1.7) | 4 (8.0) | −0.29 |
| Celiac disease | 89 (0.4) | 33 (0.3) | 0.02 | 237 (0.2) | 1 (2.0) | −0.18 |
| Crohn’s disease | 158 (0.7) | 86 (0.7) | −0.01 | 605 (0.5) | 0 | 0.10 |
| Cushing’s syndrome | 20 (0.1) | 6 (0.0) | 0.01 | 71 (0.1) | 3 (6.0) | −0.35 |
| Delirium | 11,954 (49.5) | 5,982 (49.5) | 0.00 | 16,223 (12.5) | 49 (98.0) | −3.37 |
| Depression | 8,295 (34.3) | 4,172 (34.5) | 0.00 | 20,820 (16.0) | 39 (78.0) | −1.59 |
| Hyperparathyroidism | 303 (1.3) | 150 (1.2) | 0.00 | 883 (0.7) | 1 (2.0) | −0.12 |
| Hyperthyroidism | 541 (2.2) | 279 (2.3) | 0.00 | 1,967 (1.5) | 1 (2.0) | −0.04 |
| Impaired vision | 656 (2.7) | 313 (2.6) | 0.01 | 1,117 (0.9) | 9 (18.0) | −0.61 |
| Malnutrition | 4,991 (20.7) | 2,524 (20.9) | −0.01 | 9,525 (7.3) | 21 (42.0) | −0.88 |
| Multiple sclerosis | 251 (1.0) | 120 (1.0) | 0.00 | 921 (0.7) | 2 (4.0) | −0.22 |
| Orthostatic hypotension | 2,864 (11.9) | 1,467 (12.1) | −0.01 | 4,151 (3.2) | 15 (30.0) | −0.77 |
| Osteoporosis | 3,769 (15.6) | 1,871 (15.5) | 0.00 | 11,355 (8.7) | 15 (30.0) | −0.56 |
| Ulcerative colitis | 335 (1.4) | 164 (1.4) | 0.00 | 924 (0.7) | 1 (2.0) | −0.11 |
| Vitamin D deficiency | 2,681 (11.1) | 1,369 (11.3) | −0.01 | 9,159 (7.0) | 12 (24.0) | −0.48 |
| Concomitant comedications^d^, n (%) |  |  |  |  |  |  |
| Androgen deprivation therapy | 83 (0.3) | 38 (0.3) | 0.01 | 296 (0.2) | 0 | 0.07 |
| Anticholinesterase inhibitors | 4,220 (17.5) | 2,065 (17.1) | 0.01 | 8,927 (6.9) | 19 (38.0) | −0.80 |
| Antidepressants | 7,772 (32.2) | 3,844 (31.8) | 0.01 | 30,621 (23.5) | 27 (54.0) | −0.66 |
| Benzodiazepines | 4,764 (19.7) | 2,371 (19.6) | 0.00 | 20,350 (15.6) | 23 (46.0) | −0.70 |
| Cyclooxygenase-2 inhibitors | 669 (2.8) | 314 (2.6) | 0.01 | 3,212 (2.5) | 1 (2.0) | 0.03 |
| Digoxin | 751 (3.1) | 390 (3.2) | −0.01 | 2,906 (2.2) | 6 (12.0) | −0.39 |
| Diuretics | 6,216 (25.7) | 3,098 (25.6) | 0.00 | 28,117 (21.6) | 26 (52.0) | −0.66 |
| Enzyme-inducing anticonvulsants | 839 (3.5) | 398 (3.3) | 0.01 | 5,213 (4.0) | 5 (10.0) | −0.24 |
| Glucocorticoids^e^ | 1,376 (5.7) | 695 (5.8) | 0.00 | 5,927 (4.6) | 5 (10.0) | −0.21 |
| Nonsteroidal anti-inflammatory drugs | 2,124 (8.8) | 1,058 (8.8) | 0.00 | 12,767 (9.8) | 8 (16.0) | −0.19 |
| Osteoporosis treatment | 1,183 (4.9) | 600 (5.0) | 0.00 | 6,831 (5.2) | 2 (4.0) | 0.06 |
| PD drugs^f^ | 13,354 (55.3) | 6,651 (55.0) | 0.00 | 68,262 (52.4) | 21 (42.0) | 0.21 |
| Sedatives | 1,648 (6.8) | 831 (6.9) | 0.00 | 8,221 (6.3) | 9 (18.0) | −0.36 |
| Thiazolidinediones | 346 (1.4) | 169 (1.4) | 0.00 | 2,285 (1.8) | 3 (6.0) | −0.22 |
| Health care utilization, mean (SD)^g^ |  |  |  |  |  |  |
| Number of hospitalizations | 0.5 (0.73) | 0.5 (0.71) | −0.04 | 0.2 (0.45) | 1.9 (1.76) | −1.35 |
| Number of emergency department visits | 1.2 (1.77) | 1.2 (1.61) | −0.04 | 0.4 (1.00) | 6.2 (6.53) | −1.23 |

HIV/AIDS = human immunodeficiency virus/acquired immune deficiency syndrome; PD = Parkinson disease; PDP = Parkinson disease with psychosis; SD = standard deviation.

Note: All characteristics were assessed during the entire look-back period unless otherwise stated.

^a^Patients with PD without psychosis who were selected for the matched cohort; evaluated at the index date of the matched PD diagnosis.

^b^Patients who met the criteria to enter the PDP cohort; evaluated at their first psychosis diagnosis date.

^c^ Patients who did not develop psychosis at the PD cohort eligibility date and were not selected for the matched cohort. Evaluated at their PD cohort eligibility date.

^d^Assessed in a look-back period of up to 1 year before the corresponding cohort entry/eligibility date.

^e^ Comprised all systemic glucocorticoids (excluded nonsystemic administration routes such as topical or inhaled applications).

^f^ Comprised levodopa-carbidopa, anticholinergics, dopamine agonists, monoamine oxidase B inhibitors, and catechol-O-methyltransferase inhibitors.

^g^Assessed in the 6 months before the corresponding cohort entry/eligibility date.
