# Supplementary material for: Increased risk of falls and fractures in patients with psychosis and Parkinson disease
Source: PLoS One. 2021 Jan 27;16(1):e0246121. doi: 10.1371/journal.pone.0246121 (PMC7840029; doi:10.1371/journal.pone.0246121)
Supplement: S4 Table — HIV/AIDS = human immunodeficiency virus/acquired immunodeficiency syndrome; ICD-9-CM = International Classification of Diseases, Ninth Revision, Clinical Modification; ICD-10-CM = International Classification of Diseases, Tenth Revision, Clinical Modification; PD = Parkinson’s disease; PDP = Parkinson’s disease psychosis; SD = standard deviation. Note: All characteristics were assessed during the entire look-back period unless otherwise stated. ICD-9-CM codes to identify atypical PD were 33182 and 3330. ICD-10-CM codes to identify atypical PD were G3183, G903, G239, G238, G232, G230, and G231. a Patients with PD who were selected for the matched cohort; evaluated at the index date of the matched PD diagnosis. b Patients who met the criteria to enter the PDP cohort; evaluated at their first psychosis diagnosis date. c Patients who did not develop psychosis at the PD eligibility date and were not selected for the matched cohort. Evaluated at their PD cohort eligibility date. d Assessed in a look-back period of up to 1 year before the corresponding cohort entry/eligibility date. e Comprised all systemic glucocorticoids (excluded nonsystemic administration routes such as topical or inhaled applications). f Comprised levodopa-carbidopa, anticholinergics, dopamine agonists, monoamine oxidase B inhibitors, and catechol-O-methyltransferase inhibitors. g Assessed in the 6 months before the corresponding cohort entry/eligibility date. (DOCX) [file pone.0246121.s007.docx]

**S4 Table. Sensitivity analysis of descriptive characteristics of patients with Parkinson’s disease with and without psychosis, matched cohort excluding patients with atypical Parkinson’s disease**

| **Demographic** | **PD^a^ N = 21,486** | **PDP^b^ N = 10,697** | **Standardized mean difference** |
| --- | --- | --- | --- |
| Age at index, mean (SD) | 78.2 (9.12) | 78.0 (9.37) | 0.03 |
| Sex, female, n (%) | 8,648 (40.2) | 4,278 (40.0) | 0.01 |
| **Frailty indicators, n (%)** |  |  |  |
| Ambulance/life support | 12,030 (56.0) | 6,000 (56.1) | 0.00 |
| Arthritis | 14,887 (69.3) | 7,372 (68.9) | 0.01 |
| Bladder dysfunction | 6,933 (32.3) | 3,458 (32.3) | 0.00 |
| Cancer screening | 6,319 (29.4) | 3,188 (29.8) | −0.01 |
| Coagulopathy | 1,534 (7.1) | 794 (7.4) | −0.01 |
| Dementia | 12,510 (58.2) | 6,103 (57.1) | 0.02 |
| Diabetes mellitus complications | 3,213 (15.0) | 1,632 (15.3) | −0.01 |
| Difficulty walking | 12,213 (56.8) | 6,069 (56.7) | 0.00 |
| Heart failure | 7,163 (33.3) | 3,635 (34.0) | −0.01 |
| Home hospital bed | 1,607 (7.5) | 813 (7.6) | 0.00 |
| Home oxygen | 1,328 (6.2) | 657 (6.1) | 0.00 |
| Lipid abnormality | 13,094 (60.9) | 6,564 (61.4) | −0.01 |
| Paralysis | 1,674 (7.8) | 822 (7.7) | 0.00 |
| Podiatric care | 5,373 (25.0) | 2,689 (25.1) | 0.00 |
| Rehabilitation services | 6,403 (29.8) | 3,211 (30.0) | 0.00 |
| Sepsis | 7,310 (34.0) | 3,669 (34.3) | −0.01 |
| Skin ulcer | 3,614 (16.8) | 1,839 (17.2) | −0.01 |
| Stroke/brain injury | 6,704 (31.2) | 3,348 (31.3) | 0.00 |
| Vertigo | 6,990 (32.5) | 3,430 (32.1) | 0.01 |
| Weakness | 7,256 (33.8) | 3,655 (34.2) | −0.01 |
| Wheelchair use | 2,290 (10.7) | 1,187 (11.1) | −0.01 |
| **Additional components of Charlson Comorbidity Index, n (%)** |  |  |  |
| Chronic kidney disease | 3,626 (16.9) | 1,840 (17.2) | −0.01 |
| Chronic obstructive pulmonary disease | 6,091 (28.3) | 3,019 (28.2) | 0.00 |
| Diabetes mellitus | 7,043 (32.8) | 3,543 (33.1) | −0.01 |
| Hemiplegia | 1,180 (5.5) | 585 (5.5) | 0.00 |
| HIV/AIDS | 23 (0.1) | 6 (0.1) | 0.02 |
| Leukemia/lymphoma | 522 (2.4) | 246 (2.3) | 0.01 |
| Liver disease | 724 (3.4) | 366 (3.4) | 0.00 |
| Myocardial infarction | 2,199 (10.2) | 1,095 (10.2) | 0.00 |
| Peptic ulcer disease | 698 (3.2) | 370 (3.5) | −0.01 |
| Peripheral vascular disease | 7,266 (33.8) | 3,603 (33.7) | 0.00 |
| Tumor | 4,302 (20.0) | 2,156 (20.2) | 0.00 |
| **Other predictors of falls or fractures, n (%)** |  |  |  |
| Ankylosing spondylitis | 695 (3.2) | 336 (3.1) | 0.01 |
| Celiac disease | 78 (0.4) | 26 (0.2) | 0.02 |
| Crohn’s disease | 139 (0.6) | 82 (0.8) | −0.01 |
| Cushing’s syndrome | 19 (0.1) | 6 (0.1) | 0.01 |
| Delirium | 9,967 (46.4) | 4,986 (46.6) | 0.00 |
| Depression | 7,187 (33.4) | 3,610 (33.7) | −0.01 |
| Hyperparathyroidism | 276 (1.3) | 135 (1.3) | 0.00 |
| Hyperthyroidism | 479 (2.2) | 244 (2.3) | 0.00 |
| Impaired vision | 567 (2.6) | 271 (2.5) | 0.01 |
| Malnutrition | 4,181 (19.5) | 2,173 (20.3) | −0.02 |
| Multiple sclerosis | 208 (1.0) | 101 (0.9) | 0.00 |
| Orthostatic hypotension | 2,215 (10.3) | 1,134 (10.6) | −0.01 |
| Osteoporosis | 3,315 (15.4) | 1,628 (15.2) | 0.01 |
| Ulcerative colitis | 300 (1.4) | 150 (1.4) | 0.00 |
| Vitamin D deficiency | 2,378 (11.1) | 1,214 (11.3) | −0.01 |
| **Comedications^d^, n (%)** |  |  |  |
| Androgen deprivation therapy | 71 (0.3) | 36 (0.3) | 0.00 |
| Anticholinesterase inhibitors | 3,351 (15.6) | 1,672 (15.6) | 0.00 |
| Antidepressants | 6,780 (31.6) | 3,390 (31.7) | 0.00 |
| Benzodiazepines | 4,195 (19.5) | 2,129 (19.9) | −0.01 |
| Cyclooxygenase-2 inhibitors | 610 (2.8) | 291 (2.7) | 0.01 |
| Digoxin | 682 (3.2) | 348 (3.3) | 0.00 |
| Diuretics | 5,590 (26.0) | 2,813 (26.3) | −0.01 |
| Enzyme-inducing anticonvulsants | 768 (3.6) | 370 (3.5) | 0.01 |
| Glucocorticoids^e^ | 1,128 (5.2) | 564 (5.3) | 0.00 |
| Nonsteroidal anti-inflammatory drugs | 1,929 (9.0) | 971 (9.1) | 0.00 |
| Osteoporosis treatment | 1,046 (4.9) | 545 (5.1) | −0.01 |
| PD drugs^f^ | 11,785 (54.8) | 5,901 (55.2) | −0.01 |
| Sedatives | 1,450 (6.7) | 755 (7.1) | −0.01 |
| Thiazolidinediones | 319 (1.5) | 159 (1.5) | 0.00 |
| **Health care utilization, mean (SD)^g^** |  |  |  |
| Number of hospitalizations | 0.4 (0.72) | 0.5 (0.70) | −0.04 |
| Number of emergency department visits | 1.1 (1.75) | 1.2 (1.60) | −0.05 |

HIV/AIDS = human immunodeficiency virus/acquired immunodeficiency syndrome; ICD-9-CM = *International Classification of Diseases, Ninth Revision, Clinical Modification*; ICD-10-CM = *International Classification of Diseases, Tenth Revision, Clinical Modification*; PD = Parkinson’s disease; PDP = Parkinson’s disease psychosis; SD = standard deviation.

Note: All characteristics were assessed during the entire look-back period unless otherwise stated. ICD-9-CM codes to identify atypical PD were 33182 and 3330. ICD-10-CM codes to identify atypical PD were G3183, G903, G239, G238, G232, G230, and G231.

^a^Patients with PD who were selected for the matched cohort; evaluated at the index date of the matched PD diagnosis.

^b^Patients who met the criteria to enter the PDP cohort; evaluated at their first psychosis diagnosis date.

^c^ Patients who did not develop psychosis at the PD eligibility date and were not selected for the matched cohort. Evaluated at their PD cohort eligibility date.

^d^Assessed in a look-back period of up to 1 year before the corresponding cohort entry/eligibility date.

^e^ Comprised all systemic glucocorticoids (excluded nonsystemic administration routes such as topical or inhaled applications).

^f^ Comprised levodopa-carbidopa, anticholinergics, dopamine agonists, monoamine oxidase B inhibitors, and catechol-O-methyltransferase inhibitors.

^g^Assessed in the 6 months before the corresponding cohort entry/eligibility date.
