# Supplementary material for: Increased risk of falls and fractures in patients with psychosis and Parkinson disease
Source: PLoS One. 2021 Jan 27;16(1):e0246121. doi: 10.1371/journal.pone.0246121 (PMC7840029; doi:10.1371/journal.pone.0246121)
Supplement: S5 Table — CI = confidence interval; ICD-9-CM = International Classification of Diseases, Ninth Revision, Clinical Modification; ICD-10-CM = International Classification of Diseases, Tenth Revision, Clinical Modification; IR = incidence rate; IRR = incidence rate ratio; PD = Parkinson’s disease; PDP = Parkinson’s disease psychosis; PY = person-year. Note: ICD-9-CM codes to identify atypical PD were 33182 and 3330. ICD-10-CM codes to identify atypical PD were G3183, G903, G239, G238, G232, G230, and G231. a Patients who met the criteria to enter the PDP cohort were evaluated at their psychosis diagnosis date and were matched; patients with PD without psychosis who were selected for the matched cohort were evaluated at the date of the matched PD diagnosis. b The number of events were assumed to follow a Poisson distribution. Therefore, corresponding exact 95% CIs were computed using methods described in Dobson et al. (1991). c The number of events were assumed to follow a Poisson distribution. Therefore, corresponding exact 95% CIs were computed using methods described in Sahai and Kurshid (1996). (DOCX) [file pone.0246121.s008.docx]

**S5 Table. Sensitivity analysis of incidence rates and incidence rate ratios of falls and fractures for the** **matched PD-PDP cohort^a^ excluding patients with atypical Parkinson’s disease**

| **Outcome** | **Cohort** | **Number of patients** | **Number of events** | **PYs** | **IR (95% CI)^b^ per 100 PYs** | **IRR (95% CI)^c^** |
| --- | --- | --- | --- | --- | --- | --- |
| Falls and fractures composite | PDP | 10,692 | 4,807 | 16,889 | 28.46 (27.66-29.28) | 1.44 (1.39-1.50) |
|  | PD | 21,466 | 6,708 | 33,951 | 19.76 (19.29-20.24) | Reference |
| Falls | PDP | 10,693 | 4,284 | 16,899 | 25.35 (24.60-26.12) | 1.48 (1.43-1.54) |
|  | PD | 21,469 | 5,804 | 33,968 | 17.09 (16.65-17.53) | Reference |
| Any fracture | PDP | 10,696 | 853 | 16,966 | 5.03 (4.70-5.38) | 1.18 (1.08-1.28) |
|  | PD | 21,478 | 1,455 | 34,052 | 4.27 (4.06-4.50) | Reference |
| Femur | PDP | 10,654 | 129 | 16,848 | 0.77 (0.64-0.91) | 1.13 (0.91-1.41) |
|  | PD | 21,421 | 229 | 33,870 | 0.68 (0.59-0.77) | Reference |
| Hip | PDP | 10,574 | 384 | 16,576 | 2.32 (2.09-2.56) | 1.17 (1.03-1.33) |
|  | PD | 21,289 | 664 | 33,475 | 1.98 (1.84-2.14) | Reference |
| Pelvis | PDP | 10,695 | 17 | 16,971 | 0.10 (0.06-0.16) | 1.48 (0.74-2.90) |
|  | PD | 21,483 | 23 | 34,061 | 0.07 (0.04-0.10) | Reference |
| Upper limb | PDP | 10,654 | 185 | 16,770 | 1.10 (0.95-1.27) | 1.19 (0.99-1.44) |
|  | PD | 21,412 | 312 | 33,756 | 0.92 (0.82-1.03) | Reference |
| Vertebrae | PDP | 10,690 | 17 | 16,970 | 0.10 (0.06-0.16) | 0.83 (0.44-1.50) |
|  | PD | 21,482 | 41 | 34,056 | 0.12 (0.09-0.16) | Reference |

CI = confidence interval; ICD-9-CM = International Classification of Diseases, Ninth Revision, Clinical Modification; ICD-10-CM = International Classification of Diseases, Tenth Revision, Clinical Modification; IR = incidence rate; IRR = incidence rate ratio; PD = Parkinson’s disease; PDP = Parkinson’s disease psychosis; PY = person-year.

Note: ICD-9-CM codes to identify atypical PD were 33182 and 3330. ICD-10-CM codes to identify atypical PD were G3183, G903, G239, G238, G232, G230, and G231.

^a^Patients who met the criteria to enter the PDP cohort were evaluated at their psychosis diagnosis date and were matched; patients with PD without psychosis who were selected for the matched cohort were evaluated at the date of the matched PD diagnosis.

^b^The number of events were assumed to follow a Poisson distribution. Therefore, corresponding exact 95% CIs were computed using methods described in Dobson et al. (1991).

^c^The number of events were assumed to follow a Poisson distribution. Therefore, corresponding exact 95% CIs were computed using methods described in Sahai and Kurshid (1996).
