# Supplementary material for: Increased risk of falls and fractures in patients with psychosis and Parkinson disease
Source: PLoS One. 2021 Jan 27;16(1):e0246121. doi: 10.1371/journal.pone.0246121 (PMC7840029; doi:10.1371/journal.pone.0246121)
Supplement: Attachment — Submitted filename: PONE-D-20-10989_Response_to_reviewers_20Nov2020.docx [file pone.0246121.s009.docx]

**Reviewer Comments**

**Reviewer #1: In this paper, a study is presented that examines if risk of falls and fractures differs between matched groups of people with Parkinson’s disease with and without psychosis. Large commercial insurance databases were used for this evaluation, which has some limitations as the authors mention in the discussion. The paper is well written. I have only some minor questions/suggestions:**

We appreciate the positive feedback provided.

**The term person-time needs to be explained.**

Person-time is the amount of observable time an individual or group contributes to follow-up. For example, one person followed for 2 years contributes 2 person-years; 10 people each followed for 6 months contribute a total of 5 person-years. We have added a clarification to page 6, line 108. Additionally, we have further clarified the term “immortal person-time,” which is mentioned on page 6, line 95: “(time during which a patient contributes to follow-up but has not yet met all eligibility criteria, and therefore any fall or fracture would be ineligible to be counted as an outcome).”

**The criteria mentioned in lines 129-135 are exactly the same as presented in lines 102-109. See if this can be avoided to improve readability.**

The exclusion criteria for the PDP cohort have been streamlined (page 7, line 123): “The same exclusion criteria applied at the time of PD cohort eligibility date, except the psychosis diagnosis, were applied again at the date of the psychosis diagnosis by using all available claims prior to the PDP index date.”

**People with PD before diagnosis of psychosis are considered to belong to the PD cohort. Thus, with the matching, one person can be in both groups (PD and PDP). How many people were in both groups? Does this influence the outcomes?**

Thank you for raising this good point. In the crude, unmatched population, essentially all patients in the PDP cohort were previously in the PD cohort, as shown in Figure 1. After matching, 9.2% of patients in the PD matched cohort were also in the PDP cohort. However, by design, having a fall/fracture while a patient is in the PD group, do not preclude from having a fall/fracture outcome in the PDP group. In other words, by construction, the patient’s two timepoints are independent. A patient was not allowed to match to their self. In addition, a patient was never in both cohorts at the same time. Once the patient had a psychosis diagnosis, the time in the PD cohort ended and the PDP cohort time started. So, the time periods are completely independent. Finally, when the patient entered the PDP cohort, we reevaluated their covariates at this time. So even though a patient could appear twice, we are treating them as separate patients (ie new covariates).

In summary, we consider that due to the reasons above mentioned, the fact that a patient can be in both groups had no meaningful impact of the conclusions of the study.

**Reviewer #2: This study uses administrative claims to evaluate the incidence of falls and fractures between patients with Parkinson’s disease with and without psychosis. The manuscript describes methodology in good detail and the conclusions are supported by the data. The manuscript is well written and details ethical standards that were met.**

**This report finds an increased risk of falls and fractures among matched PD and PDP patients (IRR=1.44). These findings are supported by the clinical characteristics and challenges of patients with psychosis as well as findings in previous studies. While the methodology is rigorously detailed, including great efforts to properly match PD and PDP patients based on propensity scores, a few questions remain to fully explain the outcomes.**

We appreciate the positive feedback provided.

**Recommendations to address these concerns are as follows:**

**1. Was the possibility of atypical PD considered in selection of PD patients? It is suggested to specify the specific ICD codes used. If available data permit, it would be beneficial to exclude patients with a diagnosis (initially or at any follow up visit) of MSA, PSP, DLB, etc. to avoid diagnostic misclassification.**

Thank you for this comment. We have included additional details about the specific diagnosis codes used in our case definition of PD on page 5, lines 84-87. To increase the likelihood that those identified as having PD truly do have PD, our case definition required one inpatient claim for PD, two outpatient claims for PD separated by at least 30 days but within 365 days, or one outpatient claim for PD and at least two prescription claims for PD medication.

Additionally, as recommended by the reviewer, we evaluated the potential impact of patients with atypical PD. We calculated the percentage of patients with claims for atypical PD (Lewy body dementia or diseases of basal ganglia) at baseline. The number of patients in the matched and unmatched cohorts with these claims is included below:

| **Population** | **Cohort** | **Patients with Exclusion** | **Percent** |
| --- | --- | --- | --- |
| Unmatched PD cohort with atypical PD | 154,339 | 3,371 | 2.2 |
| Unmatched PDP cohort with atypical PD | 12,132 | 1,393 | 11.5 |
| Matched PD cohort with atypical PD | 24,164 | 2,678 | 11.1 |
| Matched PDP cohort with atypical PD | 12,082 | 1,385 | 11.5 |

Based on the numbers above, we decided to include a sensitivity analysis excluding those patients with a diagnosis of atypical PD at baseline. Thus, we have described this in the methods section (page 11, line 209): “Finally, due to the possibility of PD diagnostic misclassification, we repeated the main analysis but excluded patients with a diagnosis code for atypical PD (i.e., dementia with Lewy body, multiple system atrophy, or progressive supranuclear palsy) at baseline.”

Results of this sensitivity analysis have been included as S4 table and S5 table and have been described as follows in the results section (page 19, line 314): “A total of 2,678 (11.1%) and 1,385 (11.5%) patients in the matched PD and PDP cohorts were excluded due to the co-occurrence of a diagnosis code for atypical PD (Lewy body dementia or degenerative diseases of the basal ganglia). After those exclusions, the characteristics of the remaining patients in the matched cohort were well-balanced between the PD without psychosis and PDP groups, as evidenced by the SMDs for all characteristics being near 0, between −0.05 and 0.03 (S4 Table). Comparative matched analyses excluding patients with atypical PD were almost identical to those observed in the main analysis (S5 Table); patients with PDP had higher IRRs of the composite falls/fractures outcome (IRR = 1.44; 95% CI, 1.39-1.50), falls (IRR = 1.48; 95% CI, 1.43-1.54), and fractures (IRR = 1.18; 95% CI, 1.08-1.28).”

Finally, we have added a description of this analysis in the discussion section (page 23, line 409): “The possibility of diagnostic misclassification due to the inclusion of patients with atypical PD cannot be fully rejected. Our strict PD case definition used in the present study reduced the risk of diagnostic misclassification. Moreover, main analyses were repeated excluding patients with atypical PD at baseline. The results of this sensitivity analysis were almost identical as observed in the main analysis.”

**2. In Figure 1, it is unclear where “Patients with PD and PDP diagnosis on same day n=502” fit. Should there be an arrow connecting them to the initial block of “Patients with psychosis diagnosis n=16,955”? Or elsewhere?**

Of the 154,481 patients with a PD diagnosis after inclusion and exclusion criteria, there were 502 patients who had a psychosis diagnosis on the PD cohort eligibility date. These 502 patients could not contribute to the PD cohort because of the psychosis diagnosis, but they did contribute to the PDP group because they met all the inclusion criteria for the PDP. However, these 502 patients are already included in the above box where the total of 154,481 patients are included. We have modified the text in the box “Patients with a psychosis diagnosis on the PD cohort eligibility date” and added a footnote to clarify any remaining question: “^a^ Those 502 patients with a PD diagnosis and all eligibility criteria could not contribute time to the PD without psychosis group because they were diagnosed with psychosis on the cohort eligibility date.”

**3. Although the authors stated they matched by disease trajectory, this is rather limited using administrative data. There are unknown details about diagnostic delay, progression of clinical symptoms, etc. These limitations should be discussed. It would be more appropriate to describe the matching as being based on time since PD diagnosis rather than disease trajectory.**

We appreciate the reviewer’s comment, and we agree that the exact point in the disease trajectory is difficult to define using claims data alone. First, we have modified the terminology across the documents and now use “matched based on time since cohort entry and clinical characteristics.” Additionally, we have added a new limitation in the Discussion section (page 23, line 401): “We attempted to match patients in the PD and PDP cohorts at similar disease trajectories based on time since cohort entry (i.e., first noted PD diagnosis) and relevant clinical claims. Nevertheless, the success of matching patients at similar disease trajectories is unclear due to the lack of granular clinical data in administrative claims data, such as severity of initial symptoms, progression of clinical symptoms, and other key variables.”

**4. In the group of PDP patients, was psychosis present consistently after assignment to that group or is there a possibility of transient psychosis? This should be discussed, including potential implications of inclusion of transient psychosis, if applicable.**

Following the comment of the reviewer, we have added this as a limitation (page 23, line 405): “In the same line, the possibility that a patient with transient psychosis has been classified as PDP cannot be rejected. Our eligibility criteria in the PDP cohort only required the presence of a psychosis diagnosis after the PD diagnosis without requiring repeated codes. Claims data may lack the necessary granularity to define duration or intensity of symptoms.”

**5. In the discussion, the authors raise the important points that onset of psychosis could be associated with comorbidities and that changes to antiparkinsonian medications could confound the association of PDP and falls. The authors also assert that psychosis appears to be an independent risk factor to explain the risk of falls. Although the authors addressed the potential for confounding through the use of a complex matching scheme, presenting an additional multivariate model to assess the risk of falls while controlling for disease duration, PD medication dose at time of fall, PD duration, and select comorbidities would further increase the confidence in the conclusion that psychosis is indeed an independent risk factor for falls. These results would also allow assessment of the relative contribution of each of the included factors in the risk of falls.**

We appreciate the comment of the reviewer. The matching scheme used in our analysis did control for all baseline covariates. Since separate propensity score models were constructed in each time block, and all patients’ covariates were updated at each date and used in each block. All covariates were balanced after matching as shown in Supplemental Table S3, and therefore no confounding by the measured covariates should be expected.

The objective of the current study was to evaluate the incidence rates of falls and fractures in PDP patients compared with matched PD patients rather than to build a predictive model of the risk of falls and fractures. Thus, the purpose of the covariates is to control confounding between the PD and PDP groups, which we have demonstrated through the balance of the covariates. It would be inappropriate to adjust for factors measured after the beginning of follow-up. The reviewer’s suggestion of a separate generation of a multivariate predictive model is interesting, but it is incompatible with the study design we have implemented here. The contribution of individual risk factors measured at the time of the fall was not part of our study objectives.

**With these issues addressed, this manuscript offers valuable insight into a clinically important issue. The manuscript is very well written with excellently detailed methodology, organization and writing style.**

Thank you again for the positive feedback provided.

**Journal Requirements:**

When submitting your revision, we need you to address these additional requirements.

**1. Please ensure that your manuscript meets PLOS ONE’s style requirements, including those for file naming. The PLOS ONE style templates can be found at**

<https://journals.plos.org/plosone/s/file?id=wjVg/PLOSOne_formatting_sample_main_body.pdf> and

<https://journals.plos.org/plosone/s/file?id=ba62/PLOSOne_formatting_sample_title_authors_affiliations.pdf>

We have ensured that the manuscript meets PLOS ONE’s style requirements.

**2. We note that you have provided the following information in your Ethics Statement: “Institutional review board and/or any other required reviews of the study protocol by specific committees were obtained in accordance with applicable national and local regulations.”**

**Please revise this statement to indicate whether you obtained ethics approval to carry out the study. If you did obtain ethical approval to carry out the study please include the name of the committee that provided approval. If you did not obtain ethical approval please remove this fragment.**

The ethics statement has been modified as follows:

“This is a noninterventional study using secondary data. All data used in the study were de-identified with no breach of confidentiality with regard to personal identifiers or health information. The study was reviewed by the RTI International (RTI) Institutional Review Board (IRB) and received a waiver based on a “not human research” determination. RTI holds a Federal-Wide Assurance from the Department of Health and Human Services Office for Human Research Protections that allows the organization to review and approve human subjects protocols through its IRB committees. RTI currently has three IRB committees available to review research protocols. One IRB committee is constituted to review medical research and has two members who are medical doctors. These IRBs have been audited by the US FDA and are fully compliant with applicable regulatory requirements.”

**3. We note that you have indicated that data from this study are available upon request. PLOS only allows data to be available upon request if there are legal or ethical restrictions on sharing data publicly. For information on unacceptable data access restrictions, please see** [**http://journals.plos.org/plosone/s/data-availability#loc-unacceptable-data-access-restrictions**](http://journals.plos.org/plosone/s/data-availability#loc-unacceptable-data-access-restrictions)**.**

**In your revised cover letter, please address the following prompts:**

**a) If there are ethical or legal restrictions on sharing a de-identified data set, please explain them in detail (e.g., data contain potentially identifying or sensitive patient information) and who has imposed them (e.g., an ethics committee). Please also provide contact information for a data access committee, ethics committee, or other institutional body to which data requests may be sent.**

We have added the following sentence in the cover letter: “The data were obtained pursuant to a license from a third party (IBM Watson Health). In order to provide access to the source study data, the requestor would likely need to execute an agreement from IBM Watson Health (https://www.ibm.com/products/marketscan-research-databases/purchase).”

**b) If there are no restrictions, please upload the minimal anonymized data set necessary to replicate your study findings as either Supporting Information files or to a stable, public repository and provide us with the relevant URLs, DOIs, or accession numbers. Please see** [**http://www.bmj.com/content/340/bmj.c181.long**](http://www.bmj.com/content/340/bmj.c181.long) **for guidelines on how to de-identify and prepare clinical data for publication. For a list of acceptable repositories, please see** [**http://journals.plos.org/plosone/s/data-availability#loc-recommended-repositories**](http://journals.plos.org/plosone/s/data-availability#loc-recommended-repositories)**.**

**We will update your Data Availability statement on your behalf to reflect the information you provide.**

There are restrictions to share the data.

**4. Thank you for stating the following in the Financial Disclosure section:**

**[This study was conducted by RTI Health Solutions under the direction of ACADIA Pharmaceuticals Inc and was funded by ACADIA Pharmaceuticals Inc.].**

**We note that one or more of the authors are employed by a commercial company: RTI Health Solutions and ACADIA Pharmaceuticals Inc**

**Please provide an amended Funding Statement declaring this commercial affiliation, as well as a statement regarding the Role of Funders in your study. If the funding organization did not play a role in the study design, data collection and analysis, decision to publish, or preparation of the manuscript and only provided financial support in the form of authors’ salaries and/or research materials, please review your statements relating to the author contributions, and ensure you have specifically and accurately indicated the role(s) that these authors had in your study. You can update author roles in the Author Contributions section of the online submission form.**

The authors contribution section has been updated accordingly:

JF, JBL, MEB, and MA designed the study protocol, reviewed the study results, and prepared the study manuscript. MET, CD, and GD participated in the study design and protocol, and reviewed the study results and manuscript. JB analyzed the data.

The authors JF, JBL, JB, MEB, and MA are full-time salaried employees of RTI Health Solutions, and their compensation is unconnected to the projects on which they work. MET, CD, and GD are full-time salaried employees of ACADIA, and their compensation is unconnected to the projects on which they work.

**Please also include the following statement within your amended Funding Statement.**

**“The funder provided support in the form of salaries for authors [insert relevant initials], but did not have any additional role in the study design, data collection and analysis, decision to publish, or preparation of the manuscript. The specific roles of these authors are articulated in the ‘author contributions’ section.”**

**If your commercial affiliation did play a role in your study, please state and explain this role within your updated Funding Statement.**

We have added the role of the sponsor in the updated funding statement:

“MET, CD, and GD from ACADIA contributed to the study design and protocol, and reviewed the study results and the manuscript.”

**5. Please also provide an updated Competing Interests Statement declaring this commercial affiliation along with any other relevant declarations relating to employment, consultancy, patents, products in development, or marketed products, etc.**

**Within your Competing Interests Statement, please confirm that this commercial affiliation does not alter your adherence to all PLOS ONE policies on sharing data and materials by including the following statement: “****This does not alter our adherence to PLOS ONE policies on sharing data and materials.” (as detailed online in our guide for authors** [**http://journals.plos.org/plosone/s/competing-interests**](about:blank)**). If this adherence statement is not accurate and there are restrictions on sharing of data and/or materials, please state these. Please note that we cannot proceed with consideration of your article until this information has been declared.**

**Please include both an updated Funding Statement and Competing Interests Statement in your cover letter. We will change the online submission form on your behalf.**

**Please know it is PLOS ONE policy for corresponding authors to declare, on behalf of all authors, all potential competing interests for the purposes of transparency. PLOS defines a competing interest as anything that interferes with, or could reasonably be perceived as interfering with, the full and objective presentation, peer review, editorial decision-making, or publication of research or non-research articles submitted to one of the journals. Competing interests can be financial or non-financial, professional, or personal. Competing interests can arise in relationship to an organization or another person. Please follow this link to our website for more details on competing interests:** [**http://journals.plos.org/plosone/s/competing-interests**](about:blank)**.**
